# Barriers to Screening for Abuse in Older Adults by Healthcare Providers in Rural Communities

**DOI:** 10.1093/geroni/igaf122.3136

**Published:** 2025-12-31

**Authors:** Christopher Gravett, Rebekah Perkins, Lynn Reinke, Kara Dassel

**Affiliations:** University of Utah College of Nursing, Salt Lake City, Utah, United States; University of Utah, Salt Lake City, Utah, United States; University of Utah, Salt Lake City, Utah, United States; University of Utah, Salt Lake City, Utah, United States

## Abstract

Older adult abuse is a critical yet underreported issue, leading to severe health outcomes and increased mortality. Despite its importance, screening remains inconsistent in rural healthcare settings, where providers face unique barriers. This study examines screening practices, provider confidence, and barriers in rural Southeastern Utah, a region with limited geriatric care resources. A cross-sectional survey was conducted via Qualtrics with 32 healthcare providers across settings of care. Findings indicate that 42.3% of providers rarely or never screen for abuse, despite 56.5% agreeing that screening is essential. Additionally, 52.2% are unfamiliar with standardized screening instruments, and 30.4% report low confidence in identifying abuse. Providers overwhelmingly cited lack of time, training, and uncertainty in intervention protocols as key barriers. While 72.7% were very likely to report abuse to Adult Protective Services, only 31.8% were likely to engage caregivers or family members, suggesting hesitancy in direct intervention. Qualitative insights revealed strong concerns about patient safety, a desire to improve health outcomes through the early identification and treatment of abuse-related complications; yet, many providers lacked the resources and guidance needed to act effectively. Older adult abuse screening in rural healthcare is limited, providers rarely screen patients, and the majority are unfamiliar with screening tools. Barriers include time constraints, lack of training, and unclear protocols. While providers are willing to report abuse, hesitancy in engaging caregivers highlights the need for better interventions. Improving screening requires training, standardized protocols, and policy reforms. Future research should explore training effectiveness and policy implementation to enhance detection and response.

